# Real-Time QoS Routing Protocols in Wireless Multimedia Sensor Networks: Study and Analysis

**DOI:** 10.3390/s150922209

**Published:** 2015-09-02

**Authors:** Adwan Alanazi, Khaled Elleithy

**Affiliations:** Computer Science and Engineering Department, University of Bridgeport, Bridgeport, CT 06604, USA; E-Mail: elleithy@bridgeport.edu

**Keywords:** wireless multimedia sensor network (WMSN), quality of service (QoS), routing protocols, energy efficiency, reliability, mobility, real-time

## Abstract

Many routing protocols have been proposed for wireless sensor networks. These routing protocols are almost always based on energy efficiency. However, recent advances in complementary metal-oxide semiconductor (CMOS) cameras and small microphones have led to the development of Wireless Multimedia Sensor Networks (WMSN) as a class of wireless sensor networks which pose additional challenges. The transmission of imaging and video data needs routing protocols with both energy efficiency and Quality of Service (QoS) characteristics in order to guarantee the efficient use of the sensor nodes and effective access to the collected data. Also, with integration of real time applications in Wireless Senor Networks (WSNs), the use of QoS routing protocols is not only becoming a significant topic, but is also gaining the attention of researchers. In designing an efficient QoS routing protocol, the reliability and guarantee of end-to-end delay are critical events while conserving energy. Thus, considerable research has been focused on designing energy efficient and robust QoS routing protocols. In this paper, we present a state of the art research work based on real-time QoS routing protocols for WMSNs that have already been proposed. This paper categorizes the real-time QoS routing protocols into probabilistic and deterministic protocols. In addition, both categories are classified into soft and hard real time protocols by highlighting the QoS issues including the limitations and features of each protocol. Furthermore, we have compared the performance of mobility-aware query based real-time QoS routing protocols from each category using Network Simulator-2 (NS2). This paper also focuses on the design challenges and future research directions as well as highlights the characteristics of each QoS routing protocol.

## 1. Introduction

The network routing protocols in WSNs perform similar objectives in order to distribute network reachability information. They may share the complete routing table or exchange particular information. Most existing routing approaches use dynamic information, but in some cases, static information is more suitable. However, the major objectives of introducing routing protocols for WMSNs are for prolonging the sensor network battery lifetime, ensuring the connectivity under several scenarios, enhancing the network survivability, handling energy consumption efficiently, reducing complexity and latency, improving WMSN performance, *etc.* [1,2]. Routing protocols differ due to their scalability and performance features. From another perspective, WMSNs face several restrictions due to their limited power supply and computing capability, high traffic volume and limited bandwidth [3]. There are several performance factors that affect and influence WMSN routing protocol design, such as data aggregation, network deployment, data delivery models and network dynamic. These design factors consume excess energy as well as affect the scalability and QoS.

The performance of a routing protocol is associated with an architectural design that can be dynamic or static [4]. In a dynamic network, the role of sensor nodes and sinks is very important. On the other hand, mobility of the sinks and cluster-heads is also essential. Node deployment affects the routing performance. The deployment may be self-organized or deterministic. In self-organizing deployments, the sensor nodes are randomly scattered and generate an infrastructure in an *ad hoc* fashion. In deterministic positions, the sensors are manually placed and data are forwarded through pre-defined routes. The routing protocols are based on data delivery mechanisms with respect to the reduction of energy consumption and route permanence [5,6]. Data aggregation is an issue at the routing level because sensor nodes may generate the same packets from multiple nodes that can cause the network to be flooded and waste more energy. This problem can be handled by using functions such as min, max, suppression and average. These functions can be applied partially or fully for each sensor node which leads to substantial energy savings. This technique can achieve traffic optimization and energy efficiency using well organized quality of service routing protocols [7,8].

Several efficient routing protocols in different categories have currently been introduced for the WMSNs. However, there is still the need for more research to be conducted by introducing not only energy efficient routing protocols, but also, focusing on other areas. Some important factors should be considered when developing a routing protocol such as an energy balanced network, node mobility and integration of fixed with mobile networks, and QoS [9,10,11,12].

QoS is highly important when designing routing protocols, particularly in critical applications such as healthcare and the military. Many of the introduced algorithms have been analyzed by using simulation tools *i.e.*, NS2, OPNET. Some of these algorithms might be implemented in real deployments *i.e.*, The Deadline-aware Energy-efficient Query Scheduling (DEQS) [13], Implicit Geographic Forwarding (IGF) [14] in military networks, the Energy-aware Temporarily Ordered Routing Algorithm (E-TORA) [15] in the health field. Also, there are the algorithms Two-Tier Data Dissemination (TTDD) [16], Column-Row Location, Routing On-demand Acyclic Multipath (ROAM) [17]. All of these are not completely functional in mobile environments, which can further be improved in order to control mobility and excess energy consumption. We focus in this survey paper on QoS routing protocols with a discussion of their classification, strengths and weaknesses, deployment of QoS protocols in particular applications and research directions for improving the QoS routing protocols.

## 2. Categorization and Classification of Quality of Service Routing Protocols

**Figure 1:**
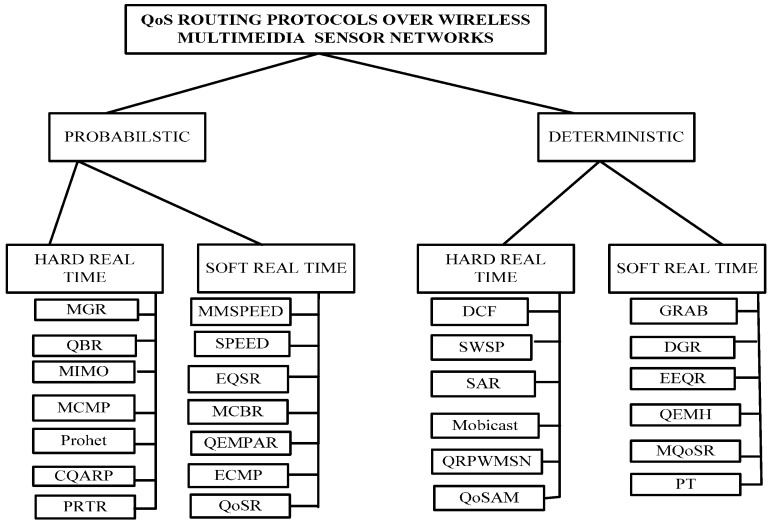
Classification of real-time quality of service (QoS) routing protocols.

Based on the research issues, we have classified QoS routing protocols into two categories, which are probabilistic and deterministic, which in trun include soft real time and hard real time QoS routing protocols. This will help researchers choose the best QoS routing protocol according to the requirements of the application in order to reduce energy consumption and obtain better throughput as given in Figure 1.

In probabilistic routing protocols, the routing between sources and destinations depends on the probability of the last lower rebroadcasted rate. In the probabilistic approach, the sensor node transmits the message with a known probability [18]. The transmission probability involves different factors such as hop-distance from source to destination, the number of hops a packet has already traveled, time in which sensor node already forwarded the packets, number of neighbor nodes, *etc.* The probability- based protocols perform directed and controlled flooding. As a result, multiple packets are copied. In addition, probabilistic protocols use the knowledge of past history. Unlike probabilistic protocols, the deterministic protocols keep the complete information of node trajectories, encounter probability of nodes and the period in which a decision is forwarded [19]. Both probabilistic and deterministic routing protocols are also classified into soft and hard real time. Soft real time protocols can miss few data points that cannot affect the performance. If some bits are missed, performance is eventually degraded. On the other hand, hard real-time protocols such as those in nuclear systems, some medical applications, military applications, avionics, *etc*, must absolutely hit every deadline.

### 2.1. Probabilistic Routing Protocols (Hard Real Time)

**Figure 2:**
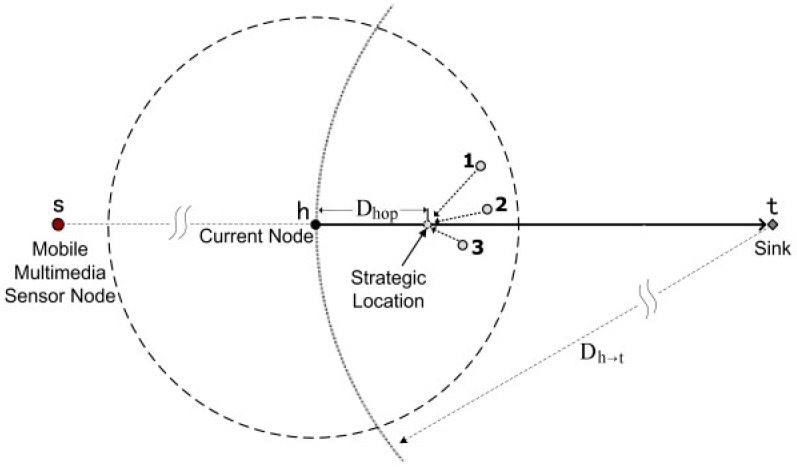
The Strategic Location Selection in MRG [20].

Multimedia Geographic Routing (MGR) introduces a new architecture called Mobile Multimedia Sensor Network (MMSN) in [20], that is based on the Mobile Multimedia Geographic Routing (MGR) scheme. In this scheme, the mobile multimedia sensor node (MMN) is used to improve the sensor network ability for event description. The purpose of this protocol is to reduce energy consumption in order to satisfy limitations on an average end-to-end delay of specific applications in MMSNs. The main goal of this protocol is to handle the delay to guarantee the priority for QoS provisioning. The protocol continuously attempts to reduce the energy consumption in order to prolong the sensor life time. This helps to exploit the energy delay adjustments for design of this protocol. However, the key operation of this protocol is to choose the suitable location of the current node for the next hop. In order to complete this, MGR estimates the distance of the desired hop for the next hop selection that can be obtained by dividing the distance between current to the sink node. MGR ensures the QoS delay and reduces about 30% energy consumption and prolongs the network lifetime as compared to classical geographic routing. MGR is more scalable than other protocols because it has the capability to control the mobility as depicted in Figure 2.

The QoS-based routing (QBR) protocol [21] is a real-hard probabilistic-based routing protocol introduced to support event and periodic-based data reporting. QBR is composed of the features of geographic routing with QoS provisioning. The data packets are forwarded in the network based on the type of the packet. QBR sets different priorities levels for each type of data packet. Thus, multiple transmission queues are introduced for handling the priorities of data packets. In addition, the node is picked based on residual energy, high link quality and the path with minimum load. The selection process of nodes consists of one-hop neighbor nodes that help reduce additional energy consumption.

In handling the congestion within the network, the ring or barrier mechanism that aggregates and captures the data packets is introduced. The barrier operation involves the barrier formation, shrinkage, repair, enlargement and termination. Despite these significant features, QBR is unable to meet the required QoS parameters. The main concern with this protocol is the use of extra control messages, which affect the throughput and consume additional energy.

Multiple Inputs and Multiple output (MIMO) is proposed in [22]. In MIMO, the data is collected in Multi-hop Virtual MIMO through multiple source nodes and transmitted to a distant sink using multiple hops. The clusters are used to organize the sensors as given in Figure 4. The cluster head transmits the data to cluster nodes that are related to a specific cluster. Additive White Gaussian Noise (WGN) is used in such a transmission with squared power path loss because of the short intracluster transmission range.

Further, the cluster nodes translate and transmit data to a cluster head to the next hop due to orthogonal Space-Time Block Code (STBC). Multi-hop Virtual MIMO shows that an average reduction of the channel between each cluster head and cluster node is estimated during construction of the clusters, so that it employs an equal Signal-to-Noise Ratio (SNR) policy to distribute the transmitted energy due to its spectral performance efficiency and simplicity.

The Multi-Constrained QoS Multi-Path routing (MCMP) protocol is proposed in [23] to handle the QoS requirements. The MCMP uses braided paths to forward the packets to the sink station, which helps maintain the QoS parameters such as end-to-end delay and reliability. The protocol structure is based on the linear integer programming, which formulates the end-to-end delay as an optimized problem. The MCMP routing algorithm builds the detailed link information for memory, sustainable computation, and overhead for the resource restricted sensor nodes. MCMP uses the local link metrics and distance to estimate the path metric. Local link metrics can help to obtain the network scalability. The goal of MCMP is to employ multiple paths to improve the network performance using moderate energy consumption. However, the protocol always prefers to choose the path that consists of the minimum number of hops to fulfill the essential QoS parameters. As a result, the protocol leads to additional energy consumption.

The Probabilistic routing protocol for Heterogeneous sensor networks (ProHet) was introduced in [24], as a probabilistic hard real-time approach that can handle asymmetric links in a dispersed fashion using local information. It uses low overhead with a guaranteed delivery rate. The working process of ProHet consists of two phases: the preparation phase that identifies the neighbor relationships and determines the reverse path for the asymmetric links, and the routing phase that selects the nodes in order to forward the message and send the acknowledgement. ProHet uses bidirectional routing abstraction to determine the reverse path for each asymmetric link. Then, it applies a probabilistic policy to select forwarding nodes based on chronological data using local information. The advantage of this protocol is to reduce energy consumption and to guarantee the delivery rate in wireless hybrid sensor networks. As with the previous QoS protocols, ProHet focuses only on hot-spot and energy consumption, as discussed in [24,25]. ProHet also lacks mobility support and decreases the throughput.

The Potential-based Real-Time Routing (PRTR) protocol is a probabilistic hard real-time QoS routing protocol introduced for making basic routing decisions [26]. The PRTR involves multiple potential fields to combine the node and queue length fields using weighing parameters. The objective of this protocol is to reduce the congestion and handle non-delay-sensitive flows to bypass the hotspots that distribute these unnecessary data packets for multipath transmission. The PRTR also utilizes calculus theory to estimate the end-to-end delay bound for a single flow. PRTR provides scalability and is suitable for large-scale WSNs by just using local information. Furthermore, PRTR satisfies the real-time routing requirements and avoid the possible congestion that causes the packet loss. However, PRTR consumes additional energy by alleviating the congestion and packet loss.

**Figure 3:**
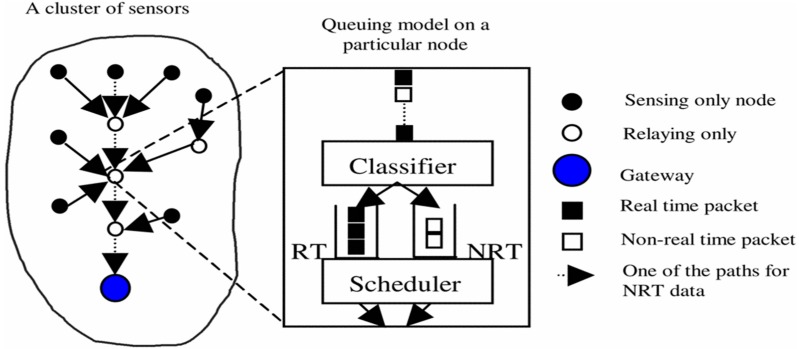
Queuing model in the cluster-based wireless sensor networks [27].

Cluster-based QoS Aware Routing Protocol (CQARP) is a probabilistic hard real-time QoS routing protocol introduced for cluster-based wireless sensor networks in [27]. This protocol employs a queuing model to tackle the non-real-time and real-time traffic. The protocol focuses on least end-to-end delay, improving the throughput and prolonging the network lifetime. The protocol involves the cost function with each link and applies the K-least cost path algorithm to determine the set of the efficient routing paths. Each path is verified against the end-to-end delay limitations. Once a path satisfies the limitations, it is selected as the path for sending the data to sink node. All the nodes are basically assigned a similar bandwidth ratio, which can create problems because some of the nodes require higher bandwidth. The strength of this protocol is to improve the throughput and prolong the network lifetime. Also, the issue of bandwidth assignment was resolved by using a different bandwidth ratio for each node. However, transmission delay was not considered, and the protocol also lacks mobility support. The working process of the protocol is depicted in Figure 3.

### 2.2. Probabilistic Routing Protocols (Soft Real Time)

The Multi-Path and Multi-SPEED (MMSPEED) Protocol is introduced to guarantee the probabilistic QoS in [28]. The QoS provisioning is done in two domains: reliability and timeliness. The reliability domain supports many reliability requirements using probabilistic multipath forwarding, and the timeliness domain can be achieved by ensuring multiple packet delivery. These mechanisms are realized in a localized manner without using global network information. The local geographic packet forwarding is improved with a dynamic benefit that compensates for local conclusion imprecision as a packet travels to its destination. The key goal of MMSPEED is to guarantee end-to-end requirements with a localized manner that supports the adaptability and scalability for large scale dynamic WSNs. It also ensures QoS differentiation in both timeliness and reliability domains. MMSPEED greatly improves both timeliness and reliability. However, MMSPEED uses greedy forwarding and geographic routing, which may not improve the performance of the network. Also, it is not a good option for long life applications because in such applications the data transmission exceeds the required energy.

The Stateless Protocol for real-time communication (SPEED) is introduced to maintain QoS for WSNs, based on soft real-time end-to-end guarantees. SPEED controls the congestion during heavy traffic load [29]. The Stateless Geographic Non-Deterministic forwarding (SNFG) routing module is used in SPEED, which works with a combination of four other modules at the network layer. SNFG maintains traffic delivery speed across a WSN using a two-tier adaptation, including traffic delivery at the networking layer and packet regulation at the MAC layer. SPEED consists of several components including Neighborhood Feedback Loop (NFL), application Programming Interface, backpressure rerouting, delay-estimation scheme, last mile processing, Nondeterministic Geographic Forwarding (NGF) algorithm, and last mile processing. SPEED consumes slightly more energy than other QoS protocols because it delivers more packets under heavy congestion. The strength of SPEED is to reduce end-to-end delay.

The energy efficient and QoS aware multi-hop routing protocol (EQSR) maximizes the network lifetime [30]. In EQSR, delayed sensitive traffic is handled and forwarded effectively to the sink node using a service differentiation concept. The goal of EQSR is to improve throughput and to reduce the end-to-end delay by using multiple paths. The protocol uses residual energy, node buffer size, and signal-to-noise-ratio for determining the best next hop. In addition, EQSR uses a data aggregation model to handle the real-time and non-real-time traffic. EQSR uses a path discovery phase that consists of initialization phase, a primary path discovery phase, and an alternative paths discovery phase.

The path discovery phase is based on directed diffusion [31]. The sink node uses multiple path discovery in order to determine the set of neighboring nodes, which are able to send the data towards the sink from the source node. Furthermore, EQSR applies a procedure of path refreshment, path selection, traffic allocation, and data transmission for maintaining the QoS and handling the different types of traffic.

Message-initiated Constrained-Based Routing (MCBR) maintains the QoS requirements [32]. MCBR is composed of explicit specifications for route constraints, QoS provisioning, and constraint-based destinations for handling the messages, and s set of QoS-based meta-schemes. The routes are set up through network flooding from the source to the destination. The data message is transmitted from the source to the destination through the route that fulfills the QoS provision for a given data message. The general purpose of a meta-data routing scheme in MCBR is to improve the end-to-end delay and throughput. In addition, MCBR involves two kinds of meta-routing strategies: search-based and constrained-flooding. However, the additional use of control packets for both types of routing strategies causes a significant overhead. In order to reduce this overhead, the QoS-aware learning-based routing protocol is proposed in [33].

Another probabilistic soft real time multi path routing protocol named QoS and Energy Aware Multi-Path Routing Algorithm (QEMPAR) was introduced to support real-time applications [34]. The goal of this protocol is to increase the network lifetime. The approach assumed that all of the nodes were randomly distributed in the intended environment. Each node was assigned unique ID. The node energy was considered equal at the beginning of the simulation. In addition, the nodes were aware of their location by using GPS and could handle the energy consumption. Based on this assumption, the nodes could communicate with other nodes beyond of their radio range. In this protocol the energy consumption model it used to determine the suitable link, path discovery, and paths assortment. In addition, the tiny packets were sent using different paths. The strength of this protocol is to prolong the network lifetime. However, throughput is affected due to increase of the latency. In addition, no mobility is considered in this protocol and using GPS makes it cost ineffective.

Energy constrained multi-path routing (ECMP) is the extension of MCMP [35]. The ECMP is proposed to frame the QoS routing problem to reduce the energy consumption. The protocol focuses on the play-back delay, reliability, and geo-spatial path selection limitations. A tradeoff between less energy consumption and the minimum number of paths is shown for improving the QoS requirements. The main purpose of driving the ECMP model is to utilize the resource constraints efficiently in order to replicate not only resourceful bandwidth utilization, but, also, insignificant energy consumption in its stringent terms. The ECMP selects those paths in the network, which satisfy the QoS requirements. However, fulfilling the QoS provisioning, routing overhead is introduced in terms of additional energy consumption and computational complexity. The overhead can affect the performance of those applications that require a certain delay and a bandwidth.

Quality-of-Service Routing (QoSR) is a deterministic soft real time protocol to determine the optimal path from the source node to base station consuming the minimum energy [36]. The nodes are particularly selected for multi-hop packet forwarding to maintain the energy efficiency. The QoSR aims to receive the successful packet to extend the network lifetime. QoSR also focused on the predefined level of reliability. The QoSR could be used in polynomial convolution with respect to the number of sensor nodes by using the Bellman-Ford algorithm. However, QoSR does not have scalability support and also does not explain how to achieve reliable data reception.

### 2.3. Deterministic Routing Protons (Hard Real Time)

Directional Controlled Fusion (DCF) protocol is introduced for data fusion and load balancing while maintaining QoS [37]. The key parameter in a multipath fusion factor provides trade-offs between multipath-expanding and multipath-converging. To guarantee the QoS for several applications, one source node is chosen as reference source per round based on some standard such as distance from the target region center, maximum remaining energy and distance to the sink. The first stage for a source node is to start a Reference-Source-Selection-Timer (RSS-Timer). A random value for each RSS-Timer is set based on specific criteria. In this phase, a small value of RSS-Timer specifies that a source has advanced admissibility as a reference source. The next step is to monitor the RSS-Timer. The source whose value terminates first is chosen as a reference source. It also broadcasts an election notification message (ENM) within the targeted region. When nodes from another source get this message, they attempt to withdraw their RSS-Timers and determine the reference source location. The next phase in the reference source is to begin the building of the reference path, and initiate the side sources attempt to transfer control packets.

Sleep/Wake Scheduling Protocol (SWSP) is introduced to preserve energy. It turns off the radio during idle time, and wakes up just before the start of the transmission of the message [38]. It uses synchronization between the sender and the receiver. Thus, nodes wake up concurrently to communicate with each other. The existing synchronization mechanism gets accurate synchronization instantaneously after exchange of synchronization messages. However there can be random synchronization faults due to non-deterministic elements in the system. A consequence of these faults is that the clock will not propagate with time and fail to match the real message transmission time. Thus, an ideal sleep/wake scheduling algorithm is introduced. It ensures a message capturing capability threshold by using less energy. Additionally, multi-hop communication is performed.

**Figure 4:**
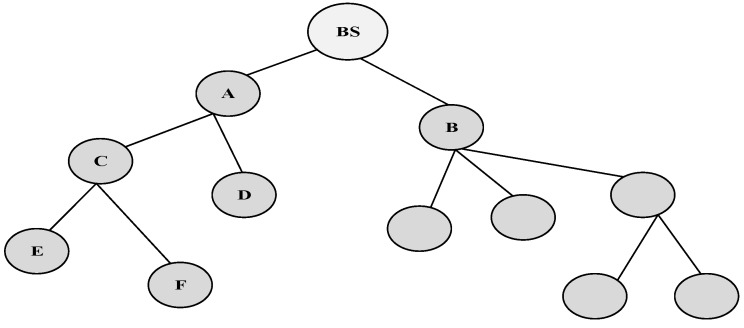
Three level cluster hierarchy [38].

The sleep/wake scheduling protocol is systematized into cluster based hierarchy, and each cluster comprises multiple cluster members and a single cluster head. The key issue of this protocol is to recognize one of the cluster members as a cluster head in one cluster. For example, “C” is cluster head of “E”, yet at the same time it is also a member of “A” as shown in Figure 4. The member nodes are synchronized during the synchronization period and the transmission period.

Sequential Assignment Routing (SAR) was the first routing protocol for WSNs that initiated the idea of QoS in routing decisions [39]. SAR decides the routing process based on three factors: (1) QoS on each path; (2) energy resources; and (3) the precedence level of each packet. SAR uses multi-path and localized path restoration techniques in order to avoid single path failure. The goal of the SAR algorithm is to reduce an average weighted QoS metric during the WSN's lifetime.

A deterministic hard real time low-complexity cooperative power provision and route planning protocol called QoS aware multi-hop routing (QoSAM) protocol was proposed for WSNs [40]. In this protocol, the sensor node use orthogonal space time block codes based on a demodulation-and-forward process. The QoSAM categorizes the QoS-aware packet forwarding problem into two disjoint processes: subsequent adaptive power allocation and dynamic programming based method planning. The goal of this protocol is to solve the QoS and energy efficiency problem for forwarding the packet using dynamic programming. Furthermore, adaptive power allocation is used for obtaining the near-optimal solution. The QoSAM aims to determine the optimized route, although energy and reliability are not fully handled.

Mobicast is the deterministic hard real-time mobile object tracking protocol introduced in [41]. The protocol uses a multi-cast routing and dynamically tracks the mobile object. In this technique, a mobile user is guided to locate the mobile object correctly without sending flooding requests to localize the mobile object. This protocol helps to preserve the energy consumption in order to prolong the network lifetime. In this approach, source and target names are used for mobile users and mobile objects respectively.

The WSN helps the source node identify the target node, and, also, keeps the tracked information of a targeted node. The approach is based on active and sleeping modes. The source node is not required to communicate to the current location of the targeted node when detecting the location. This protocol applies a face routing process explained in [42] which is based on the idea of Gabriel Graph discussed in [43] for chasing the target correctly. It also focuses on the velocity of the targeted node and its direction of movement. The protocol saves enough energy as compared with other object tracking WSN protocols. However, mobility scenarios are not completely explained and a latency issue still exists.

**Figure 5:**
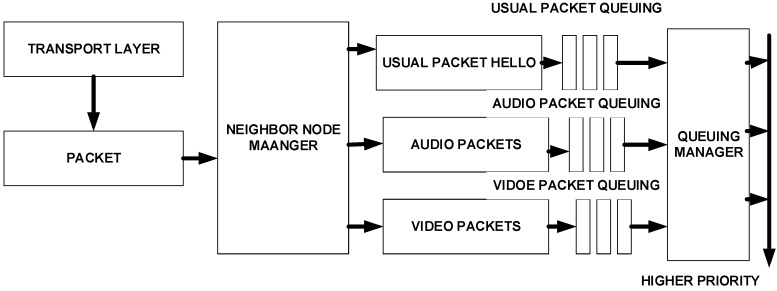
Queuing theory for proposed algorithm [44].

QoS-based routing protocol for wireless multimedia sensor network (QRPWMSN) was introduced in [44] to perform routing on each data packet according to existing QoS standards by considering the delay, energy efficiency, and reliability. This protocol is based on the geographical information model. The protocol uses a genetic algorithm and a queuing theory. QRPWMSN weights each delay in order to consume less energy by maintaining the reliability for determining the best efficient path. All the nodes are fixed and possess individual identifiers illustrated in Figure 5.

The advantage of this protocol is that it improves the transmission with less congestion However, the protocol is a bit complicated due to its use of the individual identifier that can increase the latency and reduce the throughput. In addition, it does not have mobility support and also consumes a substantial amount of energy for any transmission.

### 2.4. Deterministic Routing Protons (Soft Real Time)

GRAdient Broadcast (GRAB) is specifically designed for robust data delivery in order to control unreliable nodes and imperfect wireless links [45]. GRAB constructs and maintains the cost field by broadcasting advertisement (ADV) packets. When a node gets an ADV packet comprising the cost of a sender, it computes its cost by accumulating the link cost between sender to sender cost advertisements. The node compares this cost with the previously verified one then sets a new cost. When the node gets a smaller cost than the older one, it transmits an ADV packet containing the new cost. GRAB handles bandwidth by using an amount of credit taken in each data packet, that lets the sender regulate the strength of data delivery. The benefit of GRAB is to reply upon the communal efforts of the multiple nodes in order to distribute data without any data dependency on any individual node. However, it increases overhead by using redundant data.

The Directional Geographical Routing (DGR) Multipath routing protocol was introduced in [46]. DGR is suitable for real time video streaming over energy constrained nodes and bandwidth from a small number of detached video sensor nodes (VNs) to a sink by merging a forward error correction (FEC) coding technique. In DGR an active node VN broadcasts the packets to its direct neighbors while concatenating FEC packets of a video frame and all the data. When nodes get concatenated packets broadcast by the VN, they choose their own payload on the basis of the sequence numbers and identify the corresponding packets of nodes. Subsequently, nodes unicast the allotted packets to the sink through corresponding individual paths. In DGR, multipath routers set three paths between the source and the sink. Furthermore, each path uses a different first direct neighbor. This architecture can be efficient to route the video traffic of the network. The simulation results prove that DGR achieves a low latency equal to 0.05 ms. It also increases network lifetime and provides a better received video quality. The video peak signal to noise ratio could be improved up to 3 dB.

Energy efficient and QoS aware routing (EEQR) is a deterministic real-soft routing protocol introduced in [47]. Guaranteeing the QoS, the data prioritization is performed based on the message type. Two types of sink nodes were used: static and mobile. The static sink nodes handle the delay-sensitive messages, while mobile sinks handle the delay tolerant messages. The objective of EEQR is to improve network lifetime and coverage efficiency. In addition, it focused on the QoS parameters such as end-to-end delay, packet loss ratio, and throughput. EEQR is based on the multi-hop communication which reduces the end-to-end delay and bandwidth consumption. The packet prioritization mechanism handles the data gathering issue. Incoming traffic of the network is ranked according to the packet content significance. The proposed EEQR is divided into phases and sub-phases. The primary phases includes: setup and steady. The sub-phases comprise eight sub-phases. The setup phase involves the initialization, route update, and clustering three sub-phases. The Steady Phase consists of six sub-phases: data prioritization, data forwarding to cluster head or super node, data forwarding to static sink, mobile sink movement decision, forwarding queue weight to mobile sink, and mobile sink data gathering.

The QoS based and Energy aware Multi-path Hierarchical Routing (QEMH) protocol is introduced in [48] to fulfill the QoS requirements and energy consumption needs. QEMH is designed based on a hierarchical mechanism for consuming the minimum energy. The protocol consists of two phases. In the first phase, the QEMH selects the cluster head node based on two metrics: node distance from the sink station and residual energy of the node. In second phase, QEMH performs the route discovery process by using multiple conditions such as buffer size, residual energy, distance to sink, and signal-to-noise ratio.

Once a node detects an event, it then sends the data to the cluster head node. The responsibility of the cluster head node is to further forward it to the sink station along the paths. QEMH uses the weighted traffic allocation approach to distribute the network traffic amongst the existing paths to increase the throughput and end-to-end delay. In this approach, the cluster head node distributes the traffic between the paths based on the end-to-end delay of each path. The QEMH measures the end-to-end delay during the paths discovery process. QEMH aims to prolong the network lifetime with load-balancing that helps to balance the energy consumption uniformly throughout the network. Furthermore, QEMH deploys a queuing model to handle real-time and non-real-time traffic.

The Multi-objective QoS Routing (MQoSR) protocol was introduced [49] and is based on a geographic routing mechanism. The protocol uses a heuristic neighbor selection procedure that combines the geographic characteristics with the QoS requirements to obtain QoS improvements for several applications. The QoS provision issue for routing is articulated as path and link-based parameters. The link-based parameters are divided into delay, reliability, distance to sink, and energy consumption. The path-based parameters are presented in form of reliable data transmission, end-to-end delay, and network lifetime. MQoSR applies a different selection policy for each QoS requirement. The node selects the next hop node based on the requested requirements and the link conditions for improving the QoS provisioning. MQoSR is purely based on the on-demand routing mechanism, which makes the multiple node-disjoint paths. The MQoSR decides the cost of the each link based on link cost function, and total link cost function.

The Pheromone Termite (PT) model was introduced in [50,51] and is based on a shortest path mechanism. The protocol uses a termite-based concept to establish the routes. The protocol particularly focuses on finding the shortest path by maintaining the QoS provisions. The PT introduces two new features; pheromone sensitivity and packet generation rate. The pheromone sensitivity helps determining the link capacity prior to sending the packets over the link to avoid congestion. The packet generation rate helps inform the node regarding the number of generated packets. Both features improve the QoS provisioning, avoiding the congestion and extending the network lifetime. Another one of the best features of PT routing is to select an alternate path in case of congestion observed on the network. The PT also has support for fault-tolerance if a link is broken that is easy to maintain. The performance of PT is verified and confirmed using mathematical models and simulation.

## 3. Simulation Setup and Performance Evaluation

This section shows the performance for some of the known routing protocols; QoSR, QoSAM, MQoSR, PRTR, QEMPAR, PT and MIMO. We selected these routing protocols from each different category. The reason of selecting these real-time QoS routing protocols for comparison is that they have a mobility and query-based support. The performance of the protocols is measured using Network Simulator-2 (NS2). We used a network size of 1600 m × 1600 m.

We assume that homogenous nodes are disseminated in a flat type network. Each node initially uses 5 J of energy. The nodes are responsible for forwarding the data to the base station. The base station is located at point (0, 500). The size of the packets is 128 bytes. The residual energy of each node after six cycles is calculated in order to prolong the network lifetime. The performance analysis of the routing protocols is made using the following assumptions.

- A static sink node (base station) is set that is farther from the sensing field.
- Each node possesses uniform energy.
- Each node has a variable sensing capability and senses the field with variable rates and is also responsible for forwarding the data to a sink node.
- 50% of the nodes are mobile.
- Each sensor node has the same communication capacity and computing resources.
- The location of sensor nodes is determined prior to starting the simulation.

The remaining parameters are explained in Table 1.

**Table 1:** Simulation parameters and its corresponding values.

Parameters	Value
Size of network	1600 × 1600 m^2^
Number of nodes	450
Queue-Capacity	40 Packets
Mobility Model	Random way mobility model
Maximum number of retransmissions allowed	03
Initial energy of node	5 J
Size of Packets	128 bytes
Data Rate	300 Kb/s
Sensing Range of node	35 m
Simulation time	5 min
Average Simulation Run	06
QoS Routing Protocol	QoSR, QoSAM, MQoSR, PRTR, QEMPAR, PT and MIMO
Base station location	(0,500)
Transmitter Power	12.3 mW
Receiver Power	13.4 mW

Based on the simulation, we consider the following metrics for comparison.

- Average delivery rate
- Average energy consumption
- End-to-end delay
- Lifetime
- Bandwidth Consumption
- Packet Loss
- Deadline

### 3.1. Average Delivery Rate

**Figure 6:**
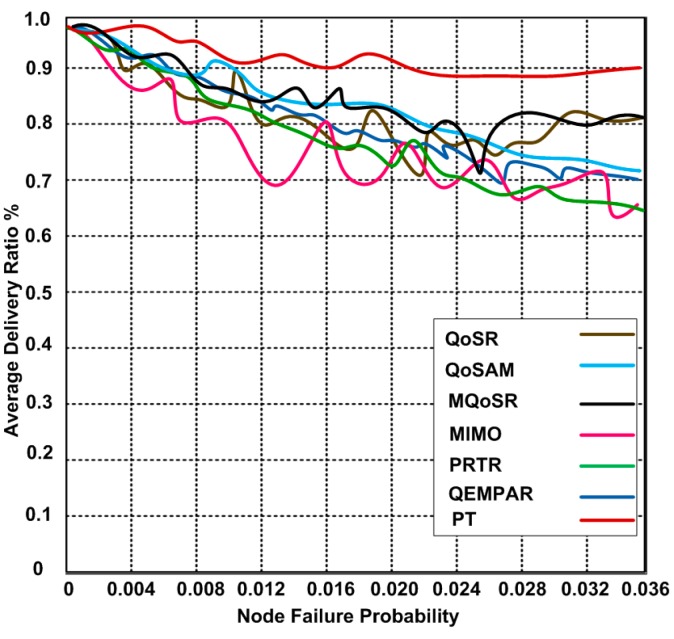
Average delivery ratios *vs.* different node failure probability.

One of the significant metrics for evaluating the performance of the routing protocols is an average delivery rate. We compare the performance using the node failure probability and an average delivery ratio as shown in Figure 6. We observe that the performance of the routing protocols is comparatively similar, but PT shows slightly higher performance than other routing protocols because PT has the support of a least distance smart search model that helps find the shortest path. We notice that these routing protocols experience problems due to node failure. As a result, the protocols show reduced performance. The reason of the reduction in the performance of the protocols is the lack of load-balancing algorithms.

### 3.2. Average Energy Consumption

**Figure 7:**
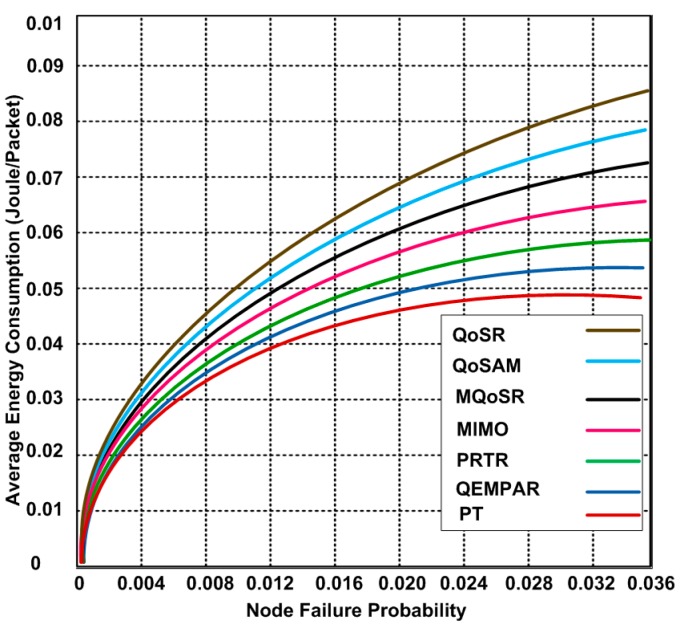
Average energy consumption *vs.* node failure probability.

We measure the energy consumption of each protocol using node failure probability. We observe that the energy consumption of each protocol increases when the node failure probability increases. However, PT show slightly lower energy consumption. The reason of the lower energy consumption for PT is the use of dynamic topology as depicted in Figure 7. The energy consumption could affect the Quality of service (QoS) provisioning. We also observe that the routing protocols consume additional energy because of node failure probability. The node failure probability could be improved using the optimized approaches.

### 3.3. End-to-End Delay

**Figure 8:**
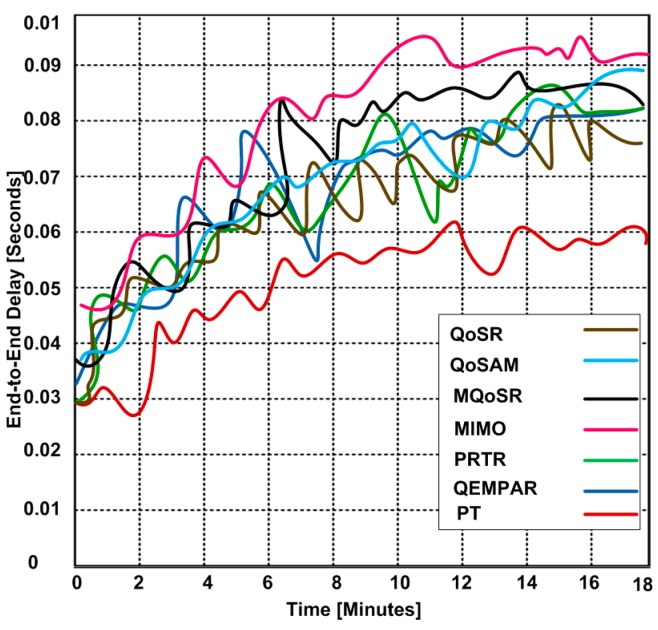
End-to-end delay of routing protocols for different time intervals.

End-to-end delay is another significant parameter for analyzing the performance of QoS-based routing protocols. We show the end-to-end delay of each routing protocol in Figure 8. Based on the results, we observe that when the time interval increases then the end-to-end delay performance of each routing protocol is affected.

In this experiment, variable packet sizes are used for the arrival rate at the sender side. One of the interesting measurements is to deal with both non-real time and real time data traffic. Finally, based on the results, we observed that PT shows slightly lower end-to-end delay as compared with other routing protocols. However, the end-to-end delay of PT can also be considered as higher within the scope of the routing performance. MIMO has higher end-to-end delay as compared with other routing protocols.

### 3.4. Lifetime

**Figure 9:**
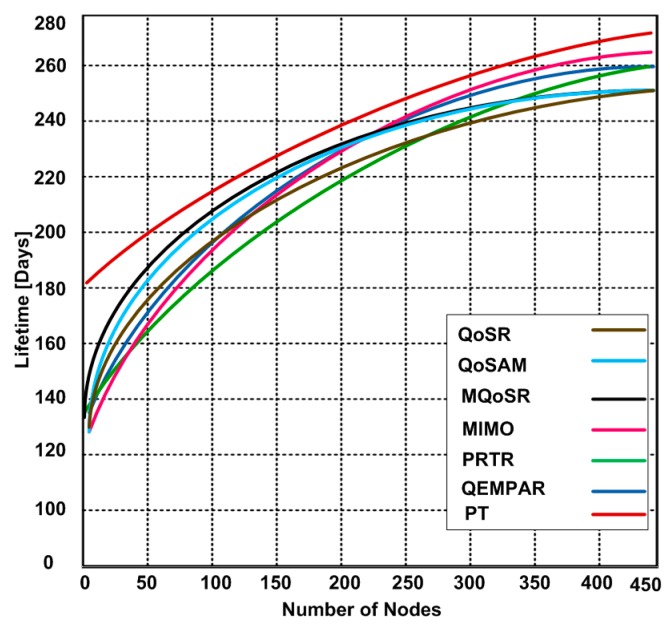
Network lifetime using different routing protocols and network size.

The primary goal of the wireless multimedia sensor networks is to improve network lifetime because sensor nodes possesses limited power and other resources. In this experiment, we measured the performance of these routing protocols using different network sizes as depicted in Figure 9.

Based on the results, we observe that the performance of each competing routing protocol is similar except for the PT routing protocol. The PT routing protocol has extended network lifetime. However, overall, the network lifetimes using these routing protocols is not encouraging. Thus, there is a dire need of optimized QoS routing protocols to prolong the network lifetime.

### 3.5. Bandwidth Consumption

**Figure 10:**
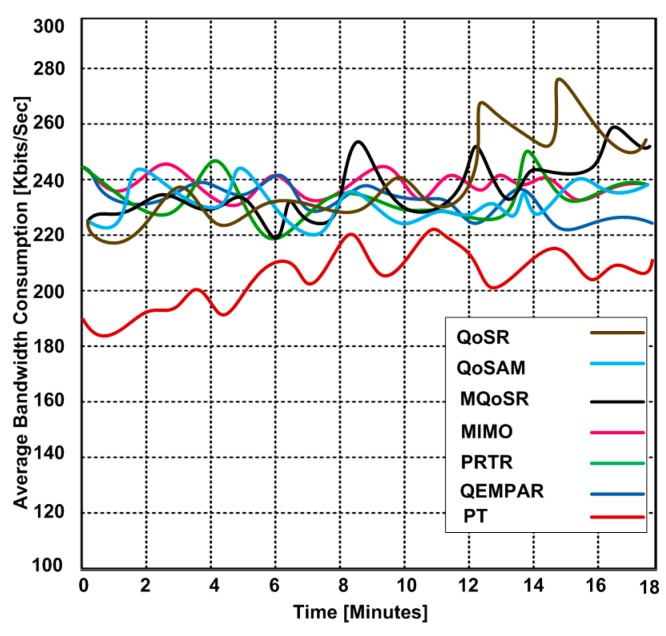
Bandwidth consumption at different time periods.

In this experiment, we want to send a file of 200 MB size. Our goal is to determine the bandwidth consumption for different real-time QoS routing protocols. Based on the simulation, we observe that all protocols have similar bandwidth consumption trends except PT which consumes less bandwidth for sending a file of 200 MB size within same amount of time. The reason for the lesser bandwidth consumption is the routing of the flow of packets through numerous different paths to the base stations. In addition, PT uses the multipath fairness solution that reallocates the network bandwidth from higher data sources to the lower data sources as long as the sensor nodes use common routing paths. Figure 10 shows the bandwidth consumption for the real-time QoS routing protocols that range from 220 Kb/s to 266 Kb/s. However, PT consumed bandwidth from 186 Kb/s to 208 Kb/s during the file-sending process. The simulation results validate that higher bandwidth consumption can affect the throughput.

### 3.6. Packet Loss

**Figure 11:**
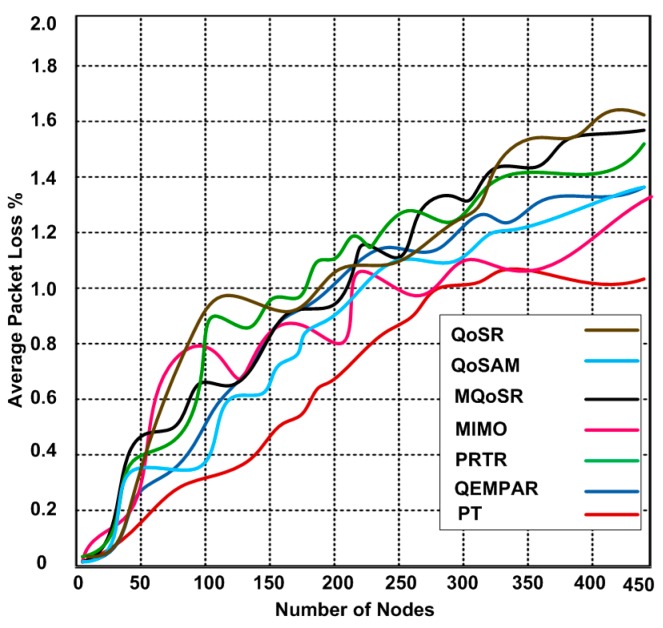
Average packet loss.

In this scenario, we determine the average packet loss for different numbers of nodes. As we can observe in Figure 11, as the number of the sensor nodes increases then the average packet loss also increases. Based on the simulation results, we confirm that the packet loss is directly proportional to the increased number of sensor nodes. Overall the average packet loss for all simulated QoS real-time routing protocols is relatively similar. However, PT produced a minimum packet loss for different numbers of nodes that ranged from 0.09% to 1.04%, while, other protocols produced 0.09%–1.62% average packet losses. QoSR, MQoSR and PRTR behave poorly with increasing numbers of sensor nodes. The reason of the maximum packet loss for these protocols is not only that they perform localized operations but also by maintaining the per-flow state, which takes a long time to recover in case of packet loss. As a result, nodes are unable to get global fairness.

### 3.7. Deadlines

**Figure 12:**
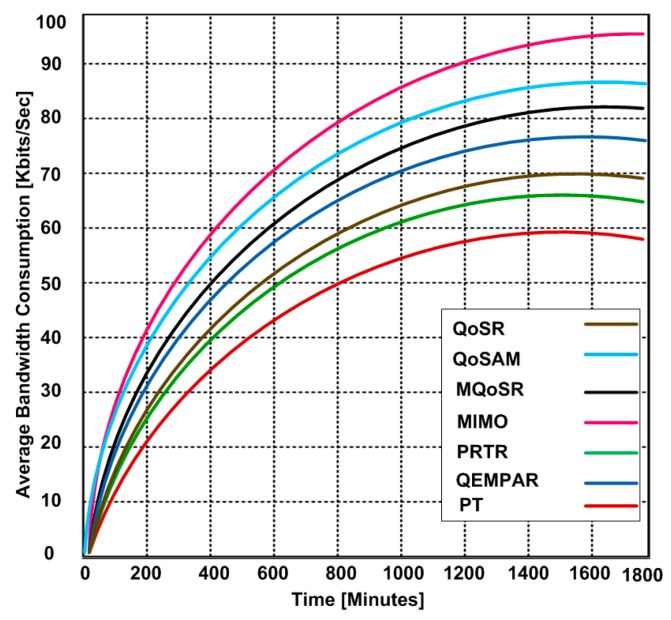
Number of miss deadlines *vs.* number of set deadlines.

In this experiment, we determine the missed deadlines that are one of the most important features to decide whether a packet is delivered within this deadline. Another goal of determining the deadline is to regulate the consumed energy for multi-hop communication. In Figure 12, we show the number of missed deadlines that helps identify the performance of the compared protocols for real time communication. Based on the simulation results, we observe that MIMO protocol missed the maximum number of deadlines as compared with other real-time QoS routing protocols. MIMO suffers from the overhead of maintaining states and routing tables at each sensor nodes and this drawback causes the maximum number of missed deadlines. The other routing protocols have similar behavior except for PT. However, when the number of set deadlines increase, then PT starts to behave positively. The path construction process in PT is simple, which causes a minimum number of missed deadlines by increasing the number of set deadlines. In addition, the path construction is divided into a single phase instead of three phases (parallel phase, growing phase and converging phases) as used by MIMO. As all these phases require additional processing time this leads to increased missed deadlines.

## 4. Discussion of the Results

Maintaining the real-time QoS parameters in the presence of mobility has been known to be one of the key challenges of WSNs and will continue to be a massive challenge for the deployment of WSNs because progression in battery technology has been slower than the progress in data communication rates and processing power. This challenge has appealed to several researchers to introduce real-time QoS protocols to balance the bandwidth consumption, packet loss, delivery rate, packet deadline, end-to-end delay and network lifetime.

To address these challenges, several routing protocols have been introduced at the network level. Real-time QoS provisioning protocols are of paramount significance because they provide real-time support, lower energy consumption and better mobility support than other categories of routing protocols. In this section, we discuss and compare the advantages and disadvantages of simulated real-time QoS routing protocols.

QoSR determines the optimal path from the source node to base station consuming the minimum energy. However, QoSR does not support scalability and also does not explain how to achieve reliable data reception. QoSAM solves the QoS and energy efficiency problem for forwarding the packet using dynamic programming. Furthermore, adaptive power allocation is used for obtaining the near-optimal solution. The QoSAM aims to determine the optimized route. However, energy and reliability are not fully handled.

MIMO is proposed for large-scaled WSNs. Multi-hop Virtual MIMO was used to reduce the channel access time between each cluster head and cluster node. It also employs an equal Signal-to-Noise Ratio (SNR) policy to distribute the transmitted energy due to its spectral performance efficiency and simplicity. As a result, MIMO reduces the energy consumption, end-to-end delay, packet loss, bandwidth consumption and improves the network lifetime. However, it behaves below standard for meeting deadlines.

The PRTR reduces the congestion and handles non-delay-sensitive flows to bypass the hotspots that distribute these unnecessary data packets for multipath transmission. The PRTR also employs calculus theory to estimate the end-to-end delay bounds for a single flow. PRTR provides scalability and satisfies the real-time routing requirements and avoid the possible congestion that causes packet loss. However, PRTR consumes additional energy while improving the congestion and packet loss.

QEMPAR increases the network lifetime. The approach assumes that all of the nodes are randomly distributed in the intended environment. Each node was assigned a unique ID. In this protocol the energy consumption model is used to determine the suitable link, path discovery, and paths assortment. The protocol prolongs the network lifetime, but throughput is affected due to the increase of the latency. In addition, mobility is considered in this protocol and the use of GPS makes it cost ineffective.

PT shows better performance than other QoS real-time routing protocols. The reason for this better performance is the use of a termite-based concept to establish the routes. The protocol particularly focuses on finding the shortest path by maintaining the QoS provisions. The PT introduces two new features: pheromone sensitivity and packet generation rate. The pheromone sensitivity helps determining the link capacity prior to sending the packets over the link to avoid congestion. The packet generation rate helps update the node regarding the number of generated packets. Both these characteristics augment the QoS, avoiding congestion and extending the network lifetime. However, PT is not suitable for small-sized networks.

It is confirmed based on the simulation results, that all existing real-time QoS routing protocols have some kind of limitations. There is a need to introduce robust routing protocols to meet the demands of several applications of MWSNs.

## 5. Challenges and Open Research Issues

Most of the existing QoS techniques only support QoS-aware routing protocols. QoS-aware routing is a crucial part for maintaining the Quality of Service framework to improve the lifetime of wireless networks. The data delivery paths are analyzed using knowledge resource accessibility along with other requirements under QoS routing schemes. There are numerous issues that are to be focused on when designing the QoS routing protocols for WMSNs. We have determined some important factors that can improve the design:

- Parameter selection (the delay, bandwidth and path computation).
- Timeliness and reliability.
- QoS state maintenance and propagation.
- Scalability and mobility.
- Maintaining the network adaptability and balancing the efficiency for low latency.

There are many introduced routing protocols, but some of them focus on maintaining QoS. In general, the main job of WSN is to sense the environment and to send the sensed data to the base station. Therefore, QoS provisioning in WMSN faces significant challenges that are discussed as follows:

▪ *Heterogeneity*: This is one of the big challenges in WMSN for maintaining the QoS because the sensors used for detecting the events are different from each other. There are some applications that require heterogeneous sensors to monitor the events and to capture images and videos of moving objects such as handling disaster situations, surveillance systems and the military battlefield environment. These applications generate data from sensors at varying rates based on different QoS limitations and delivery models. Hence, these diversified WMSNs may impose significant challenges for the provision of QoS.
▪ *Limitation of resources*: Efficient energy utilization is one of the significant challenges for maintaining the QoS. When sensor nodes are communicating they may run out of battery power. This situation can be worse when sensor nodes are underground as then they are not replaceable or rechargeable.
▪ *Bandwidth utilization*: This is generally a challenge in WSNs because WSNs involve real time and non-real time traffic. Thus, the bandwidth allocation should be maintained to balance the traffic flow between real time and non-real time communication. However, the introduction of the multimedia will pose more challenges because there will be high traffic and more bandwidth demand to process such data.
▪ *Network adaptability*: Link failures and node failures can be caused by mobility. As a result, the network topology is changed which is the issue of concern. The network encompasses the densely deployed hundreds to thousands of nodes in a landscape of interest. In this situation, a number of sensor nodes may join or leave the network which affects the QoS.
▪ *Data Redundancy*: Sensor nodes are deployed in the area of interest for sensing the situation, but most of the generated data is redundant, while this redundancy affects the reliability and fault tolerance process. As a result, a significant amount of energy is wasted. Data fusion and data aggregation are a solution to handle the redundancy. For instance, the data of sensors that generate the same image and point in the same direction can be aggregated. However, data fusion techniques and data aggregation could create problems for QoS design.
▪ *Unreliable Medium*: Radio is the communication medium in WMSNs, which is less reliable bacause of the inherent features of Medium Access Control (MAC) protocols. Furthermore, wireless links are also highly affected by several environmental factors including signal interference and noise.
▪ *Assorted Data Pattern*: This is an important issue for designing the QoS routing protocol because data can arrive in periodic and non-periodic forms. The sensing data can periodically be created in some applications at unpredictable times due to exposure to some serious events. Similarly, some sensory data can be generated at regular intervals, such as when monitoring real time environmental applications. This diversified nature of data creates significant challenges for QoS WMSN routing protocols.
▪ *Multiple Base Stations and Sinks*: Most WMSN applications involve a single base station and sink but some applications require multiple base stations and sinks, e.g., military and disaster recovery applications. In this situation, WMSNs should be capable to handle the mixed QoS levels related with multiple base stations or sinks [52]. There are several techniques available in the literature to handle such different kinds of issues to improve the QoS. However, the issue still exists that need to be resolved for QoS provisioning.

In addition to the challenges for QoS, we also focus on some of the important directions and research issues that need to be highlighted. Mobility is one of the major threats for provisioning QoS because some of the sensor network models are based on the assumption that sinks are static, but this assumption cannot be accurate for all types of scenarios. For example, battlefield scenarios consist of mixed types of sensor nodes; static and mobile. Therefore, in this situation, sink and sensor nodes should be provided with mobility support. Furthermore, the network topology also keeps on fluctuating dynamically. It is more important to address the mobility and dynamicity of WMSNs should be also considered before designing the QoS routing protocols.

The placement of heterogeneous multimedia sensor nodes is another research area for QoS provisioning [53]. Thus, secure data routing is a significant aspect that needs to be considered for WMSNs. In all of these conditions, WMSNs are highly challenging for designing QoS routing protocols. Based on our detailed survey, we have compared the characteristics of existing QoS routing protocols in Table 2.

**Table 2:** Evaluation and comparison of QoS routing protocols over WMSNs.

Routing Protocol	Energy Aware	Mobility	Scalability	Data Aggregation	Location Awareness	Query Based	Real-Time Multi-Media Support	QoS
**MMSPEED**	No	Yes	No	No	No	Yes	Yes	Yes
**ProHet**	Yes	No	No	Yes	No	Yes	No	Yes
**ECMP**	Yes	No	No	No	No	No	No	Yes
**CQARP**	Yes	No	No	No	No	Yes	No	Yes
**PT**	Yes	Yes	Yes	Yes	No	No	Yes	Yes
**QEMPAR**	Yes	No	No	No	No	Yes	No	Yes
**SPEED**	No	Yes	No	No	No	Yes	Yes	Yes
**MGR**	Yes	Yes	No	No	No	Yes	No	Yes
**SAR**	Yes	Yes	No	No	No	Yes	No	Yes
**QEMH**	Yes	No	No	No	No	No	Yes	Yes
**QRPWMSN**	No	No	No	Yes	Yes	Yes	No	Yes
**DCF**	No	No	No	Yes	Yes	Yes	No	Yes
**QBR**	No	No	No	Yes	Yes	No	Yes	Yes
**DGR**	Yes	Yes	No	No	Yes	Yes	No	Yes
**GRAB**	Yes	No	No	No	Yes	Yes	No	Yes
**SWSP**	No	No	No	Yes	No	Yes	No	Yes
**MQoSR**	No	No	No	No	Yes	No	No	Yes
**MIMO**	No	Yes	No	Yes	No	Yes	No	Yes
**Mobicast**	Yes	Yes	Yes	No	Yes	Yes	Yes	Yes
**EEQR**	Yes	No	No	No	Yes	No	No	Yes
**EQSR**	Yes	No	No	Yes	No	No	Yes	Yes
**MCBR**	Yes	No	No	No	No	No	Yes	Yes
**MCMP**	No	No	No	No	No	No	Yes	Yes
**PRTR**	No	Yes	No	No	Yes	No	Yes	Yes
**QoSR**	Yes	Yes	No	No	No	Yes	Yes	Yes
**QoSAM**	No	Yes	No	No	No	No	No	Yes

## 6. Conclusions

In this paper we have conducted a comprehensive survey of QoS routing protocols in WMSNs. The QoS routing protocols are classified into deterministic and probabilistic categories. Further, both categories are classified into soft and hard real time protocols. We have highlighted critical challenges posed by the unique features of WMSNs. In addition, we have also reviewed QoS routing protocols with strength and weaknesses in WMSNs. The paper also discusses the challenges and open research issues that will help the research community to deal with them in the future. In addition, we have simulated some known routing protocols using NS2 and also compared their performance. Finally, we have evaluated the characteristics of each routing protocol using several parameters.
